# Tenomodulin highly expressing MSCs as a better cell source for tendon injury healing

**DOI:** 10.18632/oncotarget.20495

**Published:** 2017-08-24

**Authors:** Yonghui Hou, Ming Ni, Sien Lin, Yuxin Sun, Weiping Lin, Yamei Liu, Haibin Wang, Wei He, Gang Li, Liangliang Xu

**Affiliations:** ^1^ Key Laboratory of Orthopaedics & Traumatology, The First Affiliated Hospital of Guangzhou University of Chinese Medicine, The First Clinical Medical College, Guangzhou University of Chinese Medicine, Guangzhou, P.R. China; ^2^ Department of Orthopaedics & Traumatology, Faculty of Medicine, The Chinese University of Hong Kong, Prince of Wales Hospital, Hong Kong, P.R. China; ^3^ Department of Orthopaedics, PLA General Hospital, Beijing, P.R. China; ^4^ Department of Diagnostics of Traditional Chinese Medicine, Guangzhou University of Traditional Chinese Medicine, Guangzhou, P.R. China; ^5^ Stem Cells and Regenerative Medicine Laboratory, Lui Che Woo Institute of Innovative Medicine, Li Ka Shing Institute of Health Sciences, The Chinese University of Hong Kong, Prince of Wales Hospital, Hong Kong, P.R. China; ^6^ Laboratory of Orthopaedics & Traumatology, Lingnan Medical Research Center, Guangzhou University of Chinese Medicine, Guangzhou, P.R. China

**Keywords:** mesenchymal stem cells, tenogenic differentiation, tendon, tenomodulin

## Abstract

Tendon injuries are common orthopedic problems which may cause severe morbidity. MSCs (mesenchymal stem cells) have shown promising effect on tissue engineering and have been used for the treatment of tendon injury. But the low tenogenic differentiation capacity of MSCs have hindered their application. In the present study, we have constructed the Tenomodulin (Tnmd) promoter-driven GFP expression lentiviral plasmid. After transduced into BMSCs, the expression of GFP was used to select BMSCs highly expressing Tnmd by flow cytometry. We found that MSCs with higher level of Tnmd expression had stronger tenogenic differentiation ability. Furthermore, RNA sequencing was performed to identify the molecular difference between BMSCs expressing higher and lower levels of Tnmd. And finally we demonstrated that GDF7 was upregulated in BMSCs highly expressing Tnmd and played an vital role in promoting tenogenic differentiation of BMSCs. GDF7 was mainly accounted for the elevated tenogenic differentiation ability of BMSCs with higher Tnmd expression as silencing the endogenous GDF7 significantly inhibited tenogenesis in BMSCs. In addition, the effect of BMSCs with higher Tnmd level on tendon healing was evaluated by a rat patellar tendon injury model. Taken together, our study showed that Tnmd could be used as an ideal cell surface marker to select cells with higher tenogenic differentiation ability from BMSCs, and GDF7 was indispensable for tenogenesis of MSCs.

## INTRODUCTION

Injuries to tendons and ligaments are common orthopedic problems [1]. Tendon injuries can be acute or chronic and are caused by intrinsic or extrinsic factors, either alone or in combination. It happens both in sports and in the workplace, which usually causes pain, stiffness, and loss of strength in the affected area. Achilles and patellar tendon injuries are two of most common tendon injuries in sports, which cost a big money every year worldwide [2]. Generally, the process of tendon healing occurs in three overlapping phases [3]. In the initial phase, erythrocytes and inflammatory cells, particularly neurtrophils, as well as tenocytes migrate to the wound, and type-III collagen synthesis is initiated. Then after a few days, the proliferative phase begins. About six weeks later, the remodeling phase commences, with decreased cellularity and decreased collagen and glycosaminoglycan synthesis. Although, tendon has the ability of selfheal, but the biochemical and mechanical properties of healed tendon tissue never match those of intact tendon. Up to now, one of the biggest challenges for tendon injury is to promote the healing process and improve the quality of newly formed tendon tissues.

Considerable morbidity may be produced by tendon injuries which may last for several months despite what is considered appropriate management [4]. Although many physical modalities are used in the management of tendon disorders, only a few controlled clinical trials have been performed, and most of the evidence is still pre-clinical and, at times, even controversial. As we know, extracorporeal shock wave therapy has been shown to promote healing of collagenase-induced Achilles tendinopathy in rats by inducing TGFβ1 and IGF-I expression [5]. And pulsed magnetic and electromagnetic fields with a frequency of 17 Hz improved collagen fiber alignment in a rat Achilles tendinopathy model [6]. In addition, some cytokines and growth factors such as TGFβ and IGF-I, gene therapy and tissue engineering with MSCs (mesenchymal stem cells) have been used for the treatment of tendon injury [7].

MSCs have multi-potent capacity to differentiate into a variety of cell types, including osteoblasts, adipocytes, chondrocytes, myoblasts and neurons [8, 9]. The use of MSCs for tissue repair has been reported with success [10, 11]. Tendon stem/progenitor cells is another kind of tissue-specific MSCs, and it has been identified and characterized in human, mouse and rat. These cells exhibit similar MSCs characteristics, including typical cell surface antigens, self-renewal, and multidifferentiation potential [12]. We have reported that tendon-derived stem cells could form cell sheet when treated with CTGF and ascorbic acid for 2 weeks, and finally developed into tendon-like tissue *in vivo* when transplanted into nude mouse [13]. The expression levels of tendon-related markers such as Tnmd (Tenomodulin), Scleraxis (Scx), type I collagen, etc. are much higher than that of BMSCs (bone marrow-derived MSCs) [14]. In addition, our study further demonstrate that tendon-derived stem cells could promote earlier and better tendon injury recovery [15]. However, although tendon-derived stem cells have shown promising effects on tendon healing, the outcome is still not ideal, and the most concern is that they are not practical for autologous transplantation. On the other hand, BMSCs can be easily isolated and have limited self-renewal and multilineage differentiation potentials. They also have great clinical implications, and could be used for tendon injury healing. For example, previous study has found that MSCs-collagen gel constructs could significantly improve repair biomechanics in rabbit tendon defects [16]. But their tenogenic differentiation potential is lower compared with tendon stem cells. So, it would be very interesting and necessary to find ways to improve the tenogenic differentiation ability of BMSCs. Tnmd is a well-known gene marker for the tendon and ligament lineage. The Tnmd knockout mouse showed reduced tendon cell density and proliferation without severe developmental phenotype [17]. The following study indicated that loss of Tnmd resulted in reduced self-renewal and augmented senescence of tendon stem cells [18]. In the present study, we have constructed the Tnmd promoter-driven GFP expression lentiviral plasmid. The expression of GFP was used to select BMSCs highly expressing Tnmd by flow cytometry, and evaluated their tenogenic differentiation potential *in vitro* and effect on tendon healing *in vivo* using rat patellar tendon injury model. We have proven that Tnmd could be used to select the cluster of MSCs with stronger tenogenic differentiation capacity, and identified GDF7 is an important regulator of tenogenesis. We conclude that MSCs with higher Tnmd expression level are better cell sources for tendon injury healing.

## RESULTS

### Selection of Tnmd highly expressing hMSCs by flow cytometry

In order to select MSCs highly expressing Tnmd from the hMSCs, we have constructed Tnmd promoter-driven GFP lentiviral plasmid, and transduced into hMSCs by lentiviruses. We have observed there were about 5.5% transduced hMSCs expressing GFP signal, which means Tnmd protein was highly expressed in these cells compared to the GFP negative hMSCs (Supplementary Figure 1). These GFP positive hMSCs were selected by flow cytometry, enriched and cultured for further use.

### Compare the tenogenic differentiation capacity of GFP-positive and negative hMSCs

**Figure 1:**
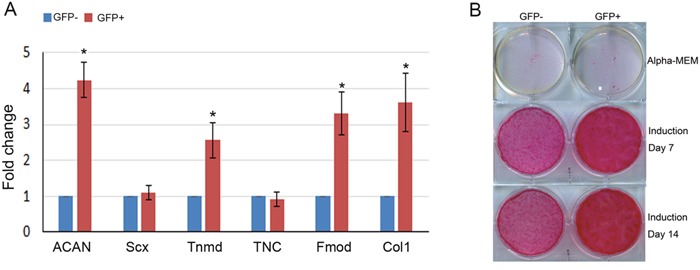
Tenogenic differentiation of GFP-positive and negative hMSCs **(A)** Total RNA was extracted from GFP-positive and negative hMSCs selected by flow cytometry. The relative expression levels of ACAN, Scx, Tnmd, TNC, Fmod and Collagen type I were evaluated by qRT-PCR. β-actin was used as an internal control. The data are expressed as mean ± SD (n=3), *p<0.05. **(B)** The MSCs were treated with tenogenic induction medium for 7 and 14 days, then Sirius Red staining was performed.

Then the expression levels of tenogenesis related marker genes in the GFP positive hMSCs were evaluated by quantitative RT-PCR, the result showed that Aggrecan (ACAN), Tnmd, Fibromodulin (Fmod) and collagen type I were significantly increased compared to the GFP negative cells, while the other markers such as Scx and Tenacin C did not show significant changes (Figure 1A). Next, these two different groups of hMSCs were subjected to tenogenic differentiation induction medium for a series of days to observe their tenogenic differentiation ability. Sirius Red staining result demonstrated that the GFP-positive hMSCs formed more collagen than the counterpart GFP-negative hMSCs at both 7 and 14 days after the induction (Figure 1B).

### Compare the gene expression profile of GFP-positive and negative hMSCs by RNAseq analysis

**Figure 2:**
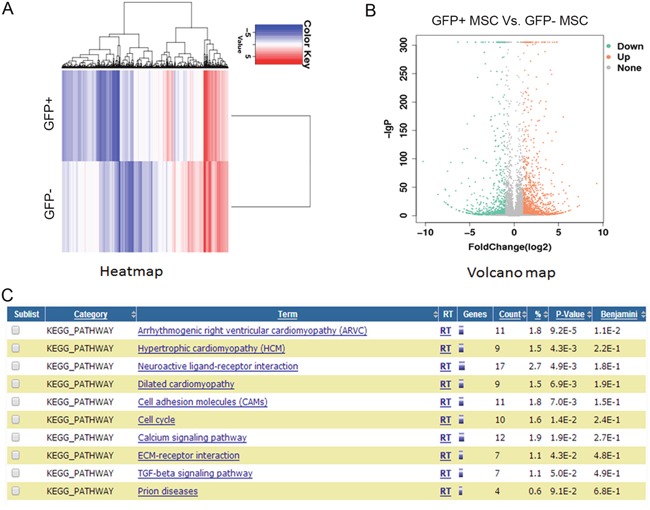
RNAseq analysis of gene expression profile of GFP-positive and negative hMSCs **(A)** Heatmap depicting expression levels of genes between GFP-positive and negative hMSCs. In total, 2800 genes were differentially expressed between GFP-positive and negative hMSCs. **(B)** Volcano map of the differentially expressed genes GFP-positive and negative hMSCs. **(C)** Top 10 enriched signaling pathways analyzed by the KEGG analysis.

**Figure 3:**
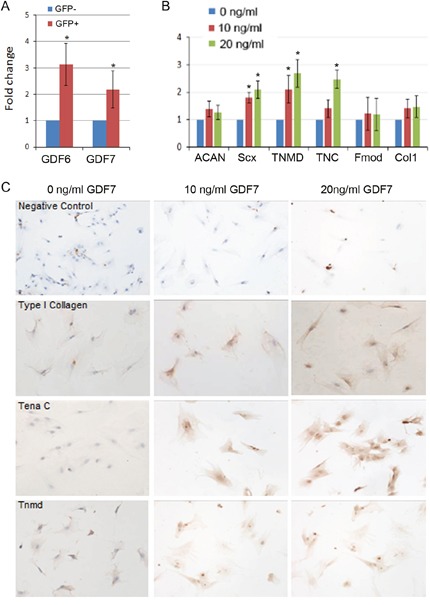
GDF7 promoted tenogenic differentiation of hMSCs **(A)** Evaluate the expression levels of GDF6 and GDF7 in GFP-positive and negative hMSCs by quantitative RT-PCR. β-actin was used as an internal control. The data are expressed as mean ± SD (n=3), *p<0.05. **(B)** GDF7 at different dosage was supplemented in the tenogenic differentiation medium for 7 days, then total RNA was extracted to evaluate the relative expression levels of ACAN, Scx, TNC, Tnmd, Fmod and Collagen type I. β-actin was used as an internal control. The data are expressed as mean ± SD (n=3), *p<0.05. **(C)** GDF7 at different dosage was supplemented in the tenogenic differentiation medium for 7 days, then immunocytochemistry staining was performed to observe the expression of Tena C, Tnmd, and Collagen type I.

In order to analyze the underlying mechanism leading to the difference of tenogenic differentiation capacity between GFP-positive and negative hMSCs, RNAseq was further performed to check the gene expression profiles of these hMSCs, respectively. The heatmap and volcano map were shown in Figure 2A & 2B. 741 up-regulated and 1124 down-regulated genes with log_2_ratio above 2 were discovered in GFP-negative hMSCs vs positive hMSCs. The KEGG (Kyoto Encyclopedia of Genes and Genomes) analysis revealed that several signaling pathways were enriched as shown in Figure 3C, among which we found that TGF-beta signaling was most attractive for further study as it was indispensable for tenogenesis and tendon development. Interestingly, we found two members of transforming growth factor beta superfamily, GDF6 and GDF7, were significantly higher in GFP- positive hMSCs.

### Effect of GDF7 on tenogenic differentiation of hMSCs

To confirm the increased levels of GDF6 and GDF7 as revealed by RNAseq, we performed quantitative RT-PCR. The result showed that GDF6 and GDF7 were increased by 2 and 3 folds in GFP positive hMSCs respectively (Figure 3A). As our previous study had already demonstrated that GDF6 could promote the tenogenic differentiation of hMSCs [19]. Then we used GDF7 at different dosages to treat hMSCs to observe its effect on tenogenic differentiation. When the hMSCs were treated with tenogenic induction medium supplemented with 10 or 20ng/ml GDF7 for 7 days, the tenogenesis-related marker genes were significantly increased by 20ng/ml GDF7, such as Scx, Tnmd and Tenascin C (Figure 3B). In addition, the immuno-staining of cells with Tnmd, Tenascin C and collagen type I antibodies further confirmed the above finding that GDF7 at dosage of 20ng/ml could increase their expression, demonstrating GDF7 could promote tenogenic differentiation of hMSCs (Figure 3C).

**Figure 4:**
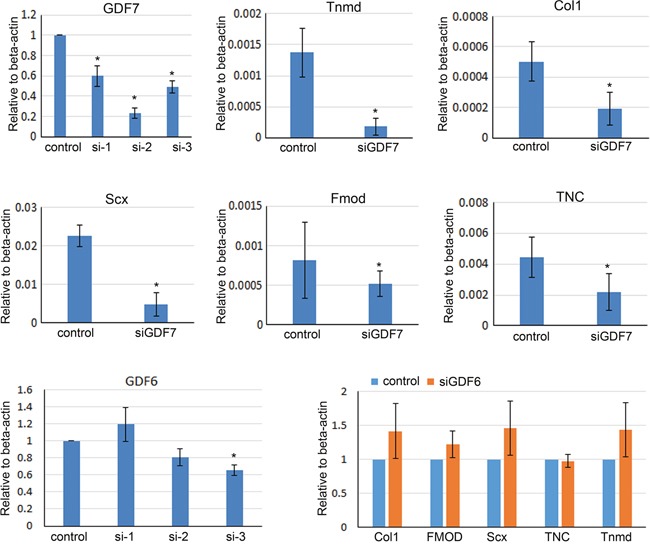
Silencing the endogenous GDF7 impaired tenogenic differentiation of hMSCs The siRNA targeting GDF6 or GDF7 was transfected into hMSCs as mentioned in Materials and Methods. siGDF7-2 and siGDF6-3 showed the best knockdown efficiency. The expression levels of Tnmd, Scx, collagen type I, Fmod and TNC were downregulated by the silencing of GDF7 (siGDF7-2). While, the silencing of GDF6 seems has no impact on tenogenic differentiation (siGDF6-3). The data are expressed as mean ± SD (n=3), *p<0.05.

In addition, the endogenous GDF6 or GDF7 was silenced using siRNA. Three different siRNAs targeting different sites of GDF6 or GDF7 were designed. And the most efficient siRNA targeting GDF6 or GDF7 was used in the following study. We found that the expression levels of Tnmd, collagen type I and Scx were downregulated by silencing of GDF7. While, the silencing of GDF6 seems had no impact on tenogenic differentiation (Figure 4). Taken together, our result showed that GDF7 was indispensable for tenogenesis.

### Ectopic tendon formation of cell sheet in nude mice

**Figure 5:**
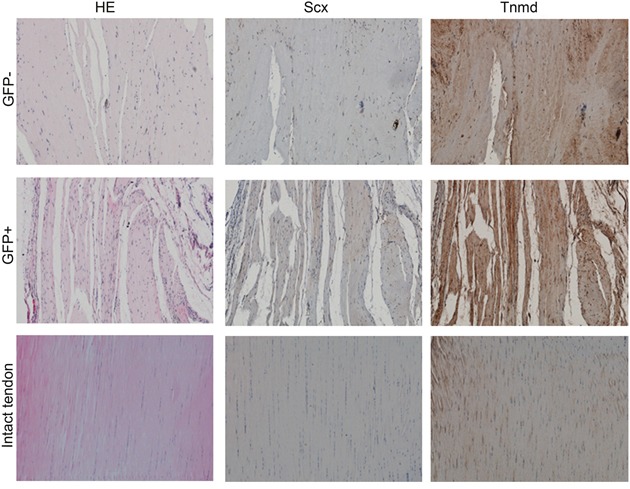
Ectopic tendon formation of cell sheet in nude mice The cell sheet formed by GFP-positive and negative hMSCs were implanted into the nude mice. After 8 weeks of transplantation, there were loosely deposited collagens in the tendon-like structure as depicted by HE staining, and more extracellular matrices and collagens were produced by GFP-positive hMSCs than that of GFP-negative hMSCs. Immunohistochemistry staining for Scx and Tnmd was also performed to observe their expression in the newly formed tendon-like tissue.

To determine the effect of Tnmd on neo-tendon formation of hMSCs *in vivo*, the cell sheet formed by GFP-positive and negative hMSCs were implanted into the nude mice. After 8 weeks of transplantation, there were loosely deposited collagens in the tendon-like structure as depicted by HE staining, and more extracellular matrices and collagens were produced by GFP-positive hMSCs than that of GFP-negative hMSCs (Figure 5). We also observed that the alignment of GFP-positive hMSCs in the newly formed tendon-like tissue was along with the collagen fibers which resembled more like the intact tendon; while in comparison, the GFP-negative hMSCs showed more randomly aligning pattern. Immunohistochemistry staining for Scx and Tnmd was also performed to observe their expression in the newly formed tendon-like tissue. And the result showed that the expression of Scx and Tnmd in the GFP-positive hMSCs formed tendon-like tissue was stronger than that of GFP-negative ones (Figure 5).

### Effect of cell sheet formed by rMSCs on tendon healing in rat patellar tendon injury model

**Figure 6:**
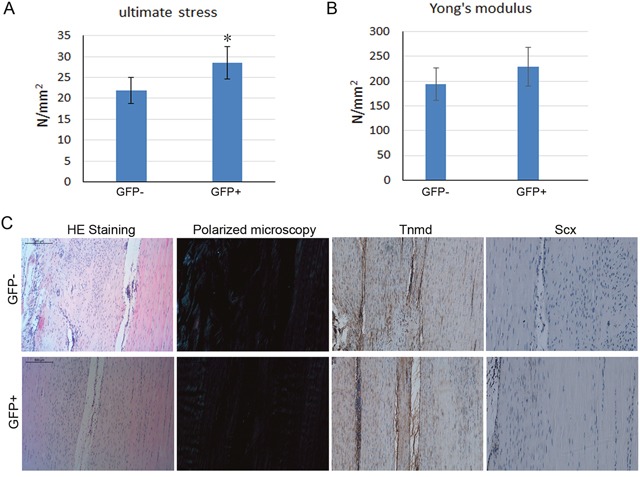
Effect of cell sheet formed by GFP positive and negative-rMSCs on tendon healing in rat patellar tendon injury model At 4 weeks after the operation, the tendon samples were collected for biomechanical testing and histological analysis. **(A-B)** The mechanical properties of the newly formed tendon tissues were analyzed by biomechanical testing. **(C)** The alignment of the collagen fibers was observed under polarizing microscope. The immunohistochemistry staining for Tnmd and Scx was also performed in the regenerated tendon tissue.

In order to evaluate the effect of Tnmd highly expressing MSCs on tendon injury healing, we enriched the GFP-positive rMSCs using same method as enriching GFP positive-hMSCs. The rMSCs were cultured to form cell sheet under the induction with CTGF and ascorbic acid. Then the rat patellar tendon injury model was established, and the cell sheet formed by GFP positive and negative-rMSCs was transplanted into the defect region. At the first week post-operation, all the skin incision wounds healed with no sign of infection, swelling and suppuration. At 4 weeks after the operation, the tendon samples were collected for biomechanical testing and histological analysis. The biomechanical testing result showed that the ultimate stress was significantly higher in the GFP-positive rMSCs formed cell sheet group compared to that in the GFP-negative group (Figure 6A); while the Young's modulus was only slightly increased in the GFP-positive rMSCs formed cell sheet group (Figure 6B). The HE staining showed that both the GFP-positive and negative group had higher cellularity in the wounded area compared with the outside normal area (Figure 6C). When it was observed under polarizing microscope, the alignment of the collagen fibers was parallel to the axes in the GFP-positive rMSCs group, while the GFP-negative rMSCs group showed a more random alignment pattern. The immunohistochemistry staining for Tnmd and Scx was also performed in the regenerated tendon tissue. And the result showed that the expression of Tnmd and Scx in the newly formed tissue was higher in the GFP-positive rMSCs group. All in all, our result showed that MSCs with higher Tnmd expression level could be differentiated into tenocytes more easily and these cells are better cell sources for tendon injury healing.

## DISCUSSION

In the present study, we successfully selected and enriched Tnmd highly expressing MSCs by using the Tnmd promoter-driven GFP reporter system. We have proved that among the heterogeneous BMSCs there was a group of MSCs with relatively stronger tenogenic differentiation capacity, and these BMSCs could be induced by ascorbic acid and CTGF to differentiate towards to tenocyte-like cells, they could form cell sheet and produce tendon-like tissues *in vivo* as well as promote tendon injury healing in a rat patellar tendon window injury model. More importantly, we found GDF7 was an indispensable regulator of tenogenesis which may regulate the expression of Tnmd in MSCs.

Tendons function as transmitters of molecular force and also provide mechanical energy storage via reversible stretching of collagen fibers [20]. The scleraxis (Scx) gene, encoding a bHLH transcription factor, is expressed in the progenitors and cells of all tendon tissues. The studies have shown that Scx is a distinct marker for tendon and ligament progenitors, and differentiated cells [21, 22]. The subsequent study by Nicholas D. Murchison et al. showed that the Scx^-/-^ knockout mice were viable but showed a dramatic disruption of tendon differentiation, the tendon matrix was reduced and disorganized, and the cellular organization of the tendons was disrupted [23]. And they surprisingly found Scx loss did not affect all tendon categories equally, that the muscle-anchoring tendons and the ligaments were not affected by Scx knockout [23]. In addition to Scx, two other transcription factors have been identified as being involved in tendon formation: the Mkx homeobox protein and the Egr1 zinc finger transcription factor. Liu and colleagues have addressed the function of Mkx in tenogenesis in mouse stem cells and in tendon repair in a mouse model for tendon injury [24]. Also, Egr1 has been shown to promote tenogenesis in stem cells and improve tendon repair in animal models of tendon injury [25, 26]. Tnmd belongs to the new family of type II transmembrane glycoproteins, which has been recognized as a tendon-specific gene marker known to be important for tendon maturation with key implications for the residing tendon stem/progenitor cells. Recent studies have found that Scx is required and sufficient for Tnmd expression. Scx deletion has been shown to lead to complete elimination of Tnmd expression, implying that Scx can directly drive Tnmd transcription [23]. Also, Egr1/2 transcription factors can induce Scx and collagen I gene expression [27], but the effect of Egr1/2 on Tnmd expression has not been reported. Taken together, we have very good reasons to believe that Tnmd may be a suitable cell surface marker for selecting the subtype of MSCs with tenogenic differentiation potential.

By using the Tnmd promoter-driven GFP reporter system, we have successfully selected and enriched Tnmd highly expressing MSCs which have also been proved to have stronger potential for tenogenic differentiation. The RNA-seq result further showed that the TGFβ (transforming growth factor beta) signaling pathway was enriched in the GFP-positive hMSCs, ie the Tnmd highly expressing hMSCs. The TGFβ superfamily and FGF (fibroblast growth factor) family are the two most important growth factors involved in tendon development and sub-sequent growth. TGFβ ligands have been extensively studied as putative candidates for accelerating tendon injury healing. For example, TGFβ ligands have been shown to activate Scx expression in mouse stem cells [28]. When tendon injury occurs, TGFβ ligands are released to recruit MSCs and other cells to take part in the repair process, and the block of TGFβ signaling in the Smad3 mutant mice would lead to the impaired tendon healing [29]. Moreover, TGFβ and FGF signaling pathways are also sufficient and required for Scx and Tnmd expression in tendons during development [30, 31]. Ectopic application of FGF-4 or FGF-8 has been shown to positively regulate the expression of Scx in the axial and limb tendon progenitors of chicken embryos [32, 33].

In the present study, we had found that GDF6 (growth/differentiation factor 6) and GDF7 were significantly increased in the GFP-positive hMSCs. The subsequent study using siRNA to silence the endogenous GDF6 or GDF7 demonstrated that GDF7 was more important than GDF6. GDF7 was indispensable for tenogenic differentiation of MSCs, while GDF6 silencing had no effect on tenogenesis although our previous study has reported that GDF6 could also promote the tenogenic differentiation of MSCs [19]. GDF6 and GDF7 belong to the TGFβ superfamily. Borjana Mikic et al. have found that GDF6 play an important role in tendon matrix modeling. They have demonstrate for the first time that a null mutation in Gdf6 is associated with substantially lower levels of tail tendon collagen content (−33%) in four-week-old male mice, which has direct functional consequences for the mechanical integrity of the tissue (45-50% reduction in material properties) [34]. GDF-7 deficient Achilles tendons contain less DNA-normalized glycosaminoglycan, and they demonstrate a shift toward a smaller collagen fibril size distribution [35], and the upregulation of GDF5 in Achilles tendons may at least in part account for the minimal phenotype observed in the Achilles tendon. Our finding is not controversial with the published reports that both GDF6 and GDF7 is indispensable for tendon development, but the *in vitro* tenogenic differentiation of MSCs result seems showed GDF7 was more important than GDF6 which was not fully consistent with the GDF6 and GDF7 deficient mice. Of course, the compensatory increase of GDF5 is one of the reasons, but other reasons are still worth for further study. All in all, all of the GDFs are believed to play a synergistic role in tendon maintenance and repair.

In our study, we used the scaffold-free cell sheet formed by BMSCs *in vivo* which could accelerate tendon injury healing, especially for the BMSCs highly expressing Tnmd. In addition, no ectopic bone formation was observed in the newly formed tendon tissue. Previous studies have found that MSCs-collagen gel constructs could significantly improve repair biomechanics in rabbit tendon defects, but ectopic bone formation was found in 28% of the rabbit tendons [16, 36]. Taken together, we have proven that Tnmd is a specific tenogenic differentiation marker which could be used to select the cluster of MSCs with stronger tenogenic differentiation capacity. Furthermore, we found GDF7 rather than GDF6 was an indispensable regulator of tenogenesis which may regulate the expression of Tnmd in MSCs. And these Tnmd highly expressing MSCs are better cell sources for tendon injury healing as demonstrated in our rat patellar tendon window injury model. Our study provides important clues to the biology of tendon and tendon injury healing that GDF7 as well as other GDFs may have great implications for accelerating tendon injury healing.

## MATERIALS AND METHODS

### Isolation and culture rat MSCs and human MSCs

All animal experiments were approved by the Animal Research Ethics Committee of the Chinese University of Hong Kong. The details of BM-MSCs isolation and culture have been described previously [37]. Briefly, the bone marrow was flushed out from the bone cavity of the Sprague-Dawley rats and subjected to density gradient centrifugation over Lymphoprep^™^ (1.077g/ml; AXIS-SHIELD, Norway) to obtain the mononuclear cells (MNCs). The MNCs were cultured in α-MEM, 10% fetal bovine serum, 2mM L-glutamine (Invitrogen, USA) at 37°C with 5% CO_2_. The medium was changed every two days. When colonies were confluent, the cells were trypsinized and re-plated for further expansion and examination. The BMSCs used in this study were between passage 3 and 8.

Human fetal bone marrow-derived MSCs (hMSCs) were donated from the Stem Cell Bank in the Prince of Wales Hospital. Human ethics approval was obtained from the Joint CUHK-NTEC Clinical Research Ethics Committee of the Chinese University of Hong Kong (Reference No. CRE-2011.383). Informed written consent form was approved by the Clinical Research Ethics Committee and signed by donor before sample collection. The hMSCs were kept in Modified Eagle's Medium of Alpha (a-MEM) (Gibco) supplemented 10% fetal bovine serum (FBS) (Gibco) and 1% penicillin/streptomycin (Gibco).

### Plasmid construction and lentivirus production

To construct lentiviral vector containing Tnmd promoter, the CMV promoter in pLentiLox 3.7 (pLL3.7) which drives GFP gene expression was cut off by NheI and NotI, and about 2kb Tnmd promoter was cloned by PCR and ligated into the plasmid. The primers used for cloning is as following: Forward primer-5’-GGGGATGAACAAGACAGGAAG-3’, Reverse primer-5’-GCAAGTGAGTCGGCTAACAGAT-3’.

Pseudo-lentivirus was produced by transient transfection of 293FT packaging cells (Invitrogen, USA) using the calcium phosphate method. Culture supernatants were harvested at 48 and 72 hours after transfection and lentiviral particles were concentrated using PEG6000 [38]. For transduction, 1×10^5^ cells were seeded into 6-well plate and incubated with lentivirus and 8 μg/mL polybrene in the incubator for 24h.

### Tenogenic differentiation of BMSCs

BMSCs at passage 3 were plated at 5000 cells/cm^2^ in 6-well plates and 24-well plates and cultured in complete culture medium until the cells reached confluence. The cells were then cultured with low glucose Dulbecco's Modified Eagle Medium (LG-DMEM) supplemented with ascorbic acid (25μM) (Catalog#A-0278, Sigma, USA) and CTGF (25 ng/ml) (Human CTGF, Catalog#120-19, PeproTech, USA) at 37°C, 5% CO_2_. The medium was changed every 3 days. After 2 weeks, the mRNA expression of tendon specific markers was examined by quantitative real time RT-PCR (qRT-PCR).

### RNA extraction and quantitative real-time PCR

The cells were harvested and homogenized for RNA extraction with RNeasy mini kit (Qiagen, Hilden, Germany). The mRNA was reverse-transcribed to cDNA by the PrimeScript First Strand cDNA Synthesis Kit (Takara). 5μl of total cDNA of each sample were amplified in a final volume of 25μl of reaction mixture containing Platinum SYBR Green, qPCR SuperMix-UDG ready-to-use reaction cocktail and specific primers using the ABI StepOne Plus system (all from Applied Biosystems, CA, USA). The expression of target gene was normalized to that of β-actin gene which was shown to be stable in this study. Relative gene expression was calculated with the 2^-ΔCT^ formula. The sequences of the primers were shown in Supplementary Table 1. Primer sequences were determined through established GenBank sequences.

### RNA-seq and data analysis

Total RNA was obtained from the GFP-positive and negative hMSCs using TRIzol Reagent (Takara, Dalian, China). The quality and integrity of total RNA samples were assessed using a 2100 Bioanalyzer or a 2200 TapeStation (Agilent Technologies) according to the manufacturer's instructions. The preparation of whole transcriptome libraries and deep sequencing were performed by the Annoroad Gene Technology Corporation (Beijing, China). The RPKM from RNAseq has been uploaded in the Supplementary Data. DAVID bioinformatics tool was also used for functional annotation enrichment and clustering.

### Cell transfection

The siRNAs targeting GDF6 and GDF7 were designed and synthesized by Genepharma Company (Shanghai, China). The sequence of each siRNA was listed in Supplementary Table 2. siRNA transfection was performed with Lipofectamine 3000 transfection reagent (Thermo, USA) according to the manufacturer's instructions. Briefly, hMSCs were plated in a-MEM complete medium into 6-well plate, and allowed to reach 50–60% confluency. Three days later the total RNA was extracted for real time PCR analysis.

### *In vivo* neo-tendon formation by engineered scaffold-free tendon tissue in nude mice

In order to demonstrate the GFP-positive and negative MSCs can form neo-tendon tissue *in vivo*, the cell sheets formed by these two types of cells were transplanted to the dorsal sites of nude mice. Briefly, total 8 mice were used; after anesthesia, an incision was made on the dorsum and a subcutaneous pocket was created to expose posterior midline. The cell sheet was sutured to posterior midline at both ends using Ethicon 6-0 suture, there was tensile strength on the tendon graft with the mice movement. After 8 weeks, the implanted tissues were harvested, subject to *ex vivo* histological analysis for examination of vascularity and collagen fiber alignment.

### Patellar tendon injury and repair animal model

Twelve Sprague Dawley male rats (8 weeks old, body weight of 250-300g) were used in this study. To create the tendon defect, the central one-third of the patellar tendon (1.5 mm in width) was removed from the distal apex of the patella to the insertion of the tibia tuberosity with two stacked sharp blades according to our well-established protocol. The operated rats were divided into 2 groups: (a) GFP-positive rMSCs cell sheet group and (b) GFP-negative rMSCs cell sheet group. The engineered scaffold-free tendon tissue was placed in the tendon defect and sutured to the patellar bone and tibia tuberosity using Ethicon 6-0 suture. The animals were allowed to have free-cage activity until euthanasia. At week 4 after surgery, the animals in each group were killed and the patellar tendons were harvested for *ex vivo* histological examination and biomechanical test.

### Biomechanical testing

We followed the procedures as described in previous study [39]. The patellar tendon-tibia composite was first isolated. The regenerated tissue in the window wound connected to the bony ends was then isolated by excising the medial and lateral healthy tendon using two stacked blades similar to the creation of tendon defect. The composite was fixed on a custom-made testing jig with two clamps. The lower one was used to fix the tibia shaft and plateau while the upper one was used to fix the proximal patella, the quadriceps muscles and its tendons without creating mechanical stress to the junction and the mid-substance. The whole construct was then mounted onto the Hounsfield H25KS mechanical testing machine (Tinius Olsen Ltd, Salfords, UK). The test to failure was performed at a testing speed of 40 mm/min and preload of 0.1N using a 50-N load cell. The ultimate stress (N/mm^2^) was calculated based on the ultimate load divided by the cross-sectional area at the break point measured by high-resolution Vevo 770 animal ultrasound system (Visualsonics, Toronto, Canada) with images taken immediately prior to the biomechanical test. The Young's modulus (N/mm^2^) was calculated from the linear slope of a stress strain curve.

### Histology and immunohistochemistry

The formed neo-tendon tissue and regenerated patellar tendon tissue were washed in PBS, fixed in buffered formalin and 70% ethanol, embedded in paraffin and sectioned for staining with hematoxylin and eosin. Immunohistochemistry was done as described previously [39]. Briefly, after deparaffination, the sections were rehydrated, quenched of endogenous peroxidase activity and subject to antigen retrieval. After blocking with 5% normal donkey and goat serum, the sections were incubated with specific antibodies against Tnmd and Scx (sc-98875 and sc-87425, Santa Cruz Biotechnology, CA, USA) at dilution of 1:100 at 4°C overnight. Goat anti-rabbit horseradish peroxidase (HRP)-conjugated secondary antibody and donkey anti-goat horseradish peroxidase (HRP)-conjugated secondary antibody (Santa Cruz Biotechnology, CA, USA; dilution 1:100) were then added for 30min respectively. Afterward, the sections were rinsed, counterstained in hematoxylin, dehydrated with graded ethanol and xylene, and mounted with p-xylene-bis-pyridinium bromide (DPX) permount (Sigma Aldrich, St Louis, MO, USA). All incubation times and conditions were strictly controlled. The sections were examined under light microscopy (DMRXA2, Leica Microsystems Wetzlar GmbH, Germany).

### Data analysis

All data were presented as mean ± SD and Statistical analysis was performed using one-way analysis of variance (one-way ANOVA). A value of P < 0.05 was considered statistically significant.

## SUPPLEMENTARY MATERIALS FIGURES AND TABLES




