# Supplementary material for: Tenomodulin highly expressing MSCs as a better cell source for tendon injury healing
Source: Oncotarget. 2017 Aug 24;8(44):77424–35. doi: 10.18632/oncotarget.20495 (PMC5652790; doi:10.18632/oncotarget.20495)
Supplement: Supplementary file 1 [file oncotarget-08-77424-s001.pdf]

Tenomodulin highly expressing MSCs as a better cell source for tendon injury healing

SUPPLEMENTARY MATERIALS

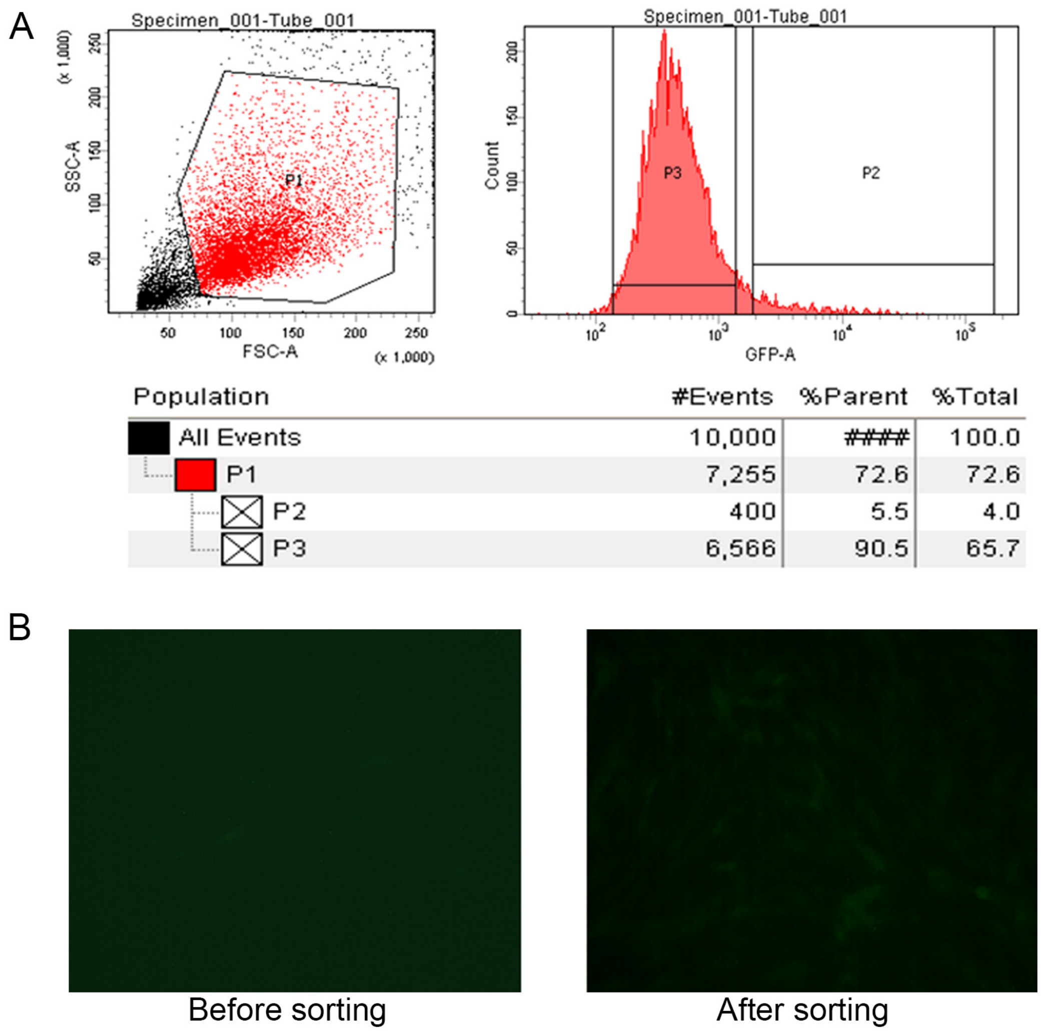

Population	#Events	%Parent	%Total
All Events	10,000	####	100.0
P1	7,255	72.6	72.6
P2	400	5.5	4.0
P3	6,566	90.5	65.7

Supplementary Figure 1: Selecting Tnmd highly expressing hMSCs by flow cytometry.

Supplementary Table 1: Sequences of primers for real time PCR

Gene name	*Forward primer sequence (5' to 3')*	*Reverse primer sequence (5' to 3')*
ACAN	GCGAGTTGTCATGGTCTGAA	GCCGGCCCAAGAGAGAA
Scx	CTGGCCTCCAGCTACATCTC	CGGTCCTTGCTCAACTTTCT
Tnmd	TGCTGTAGAAAGTGTGCTCCA	GATTTGTGGACTGGTGTTTGG
TNC	GGCTCTAGGGCTCTAGGGATT	CCTAGGTCTCTCGCCCATC
GAPDH	CGTAAAGACCTCTATGCCAACA	CGGACTCATCGTACTCCTGCT
FMOD	AGAAGTTCACGACGTCCACC	CAGCCTCCTTGAGCTAGACC
Col1	CACTGGTGATGCTGGTCCTG	CGAGGTCACGGTCACGAAC
GDF6	TGCACGTGAACTTCAAGGAG	CCCGCGTCGATGTATAGAAT
GDF7	TCATTCAGACGCTGCTCAAC	GTGTGCTCTCCCTCCTCTGA

Supplementary Table 2: Sequences of siRNA targeting GDF6 and GDF7

Gene	Sequence	
	Sense (5'-3')	Antisense (5'-3')
siGDF7-1	GCAGAGGAAAGAGAGCUUATT	UAAGCUCUCUUUCCUCUGCTT
siGDF7-2	CCACCAACCAUGCCAUCAUTT	AUGAUGGCAUGGUUGGUGGTT
siGDF7-3	CCAACAACGUUGUCUACAATT	UUGUAGACAACGUUGUUGGTT
siGDF6-1	CUGGUGGUAUUCACCAGAUTT	AUCUGGUGAAUACCACCAGTT
siGDF6-2	CCCAUCAGCAUUCUAUACATT	UGUAUAGAAUGCUGAUGGGTT
siGDF6-3	CGGGCAAUAAUGUGGUCUATT	UAGACCACAUUAUUGCCCGTT

Supplementary Data: RPKM result of RNAseq

See Supplementary File 1
